# Retraction: Glycinebetaine mitigates drought stress-induced oxidative damage in pears

**DOI:** 10.1371/journal.pone.0277597

**Published:** 2022-11-16

**Authors:** The PLOS ONE Editors

The *PLOS ONE* Editors retract this article [1] because it was identified as one of a series of submissions for which we have concerns about authorship, competing interests, and peer review. We regret that the issues were not addressed prior to the article’s publication.

TN, TZ, YQ, PW, DAAB, MSAH, XG, and XY did not agree with the retraction. GZ and EL either did not respond directly or could not be reached.

## References

[1] Niu T, Zhang T, Qiao Y, Wen P, Zhai G, Liu E, et al. (2021) Glycinebetaine mitigates drought stress-induced oxidative damage in pears. PLoS ONE 16(11): e0251389. 10.1371/journal.pone.0251389 34793480 PMC8601463

